# Acute Hepatitis C in HIV-1 Infected Japanese Cohort: Single Center Retrospective Cohort Study

**DOI:** 10.1371/journal.pone.0100517

**Published:** 2014-06-19

**Authors:** Masahiro Ishikane, Koji Watanabe, Kunihisa Tsukada, Yuichi Nozaki, Mikio Yanase, Toru Igari, Naohiko Masaki, Yoshimi Kikuchi, Shinichi Oka, Hiroyuki Gatanaga

**Affiliations:** 1 AIDS Clinical Center, National Center for Global Health and Medicine, Tokyo, Japan; 2 Department of Gastroenterology, National Center for Global Health and Medicine, Tokyo, Japan; 3 Pathology Division of Clinical Laboratory, National Center for Global Health and Medicine, Tokyo, Japan; 4 Research Center for Hepatitis and Immunology, National Center for Global Health and Medicine, Tokyo, Japan; 5 Field Epidemiology Training Program Japan, Infectious Disease Surveillance Center, National Institute of Infectious Diseases, Tokyo, Japan; 6 Global Infectious Diseases of Infection and Epidemiology, Medical Sciences Doctoral Program, Graduate School of Medicine, Tohoku University, Miyagi, Japan; 7 Center for AIDS Research, Kumamoto University, Kumamoto, Japan; University of Sydney, Australia

## Abstract

**Objectives:**

HCV co-infection is a poor prognostic factor in HIV-1-infected patients. Although the number of newly reported patients who show seroconversion is increasing, the clinical features are still unclear, especially in Asian countries.

**Design:**

A single-center retrospective cohort study of patients diagnosed between 2001–2012.

**Methods:**

Acute hepatitis C (AHC) was diagnosed upon detection of high serum ALT (>100 IU) followed by anti-HCV seroconversion. Clinical characteristics, HIV-1-related immunological status and IL-28B genotypes (rs12979860, rs8099917) were collected. We compared these variables between patients with and without spontaneous clearance of HCV and between responders and non-responders to treatment with pegylated interferon (PEG-IFN) plus ribavirin.

**Results:**

Thirty-five patients were diagnosed with AHC during the study period. The majority (96.9%) were MSM. Three were lost to follow-up. Seventy-five percent of patients with AHC (24/32) were asymptomatic and found incidentally to have high serum ALT. Compared to those who did not show spontaneous clearance, patients with spontaneous HCV viral clearance showed more symptoms and more severe abnormalities related to acute hepatitis. Spontaneous clearance was seen in 4 out of 28 patients with CC+TT genotype, but not in 6 patients with IL-28B CT+TG genotype. PEG-IFN plus ribavirin treatment was initiated in 12 out of 28 cases without spontaneous clearance. The sustained virological response rate was high (81.8%, 9/11), even in cases with CT+TG genotype infected with HCV genotype 1b (SVR 2/2).

**Conclusions:**

Careful attention to AHC is needed in HIV-1-infected MSM. Early diagnosis and PEG-IFN plus ribavirin treatment should be considered for AHC cases.

## Introduction

The estimated worldwide prevalence of hepatitis C virus (HCV) infection is 2–3% [1]. HCV co-infection increases morbidity rate in HIV infected individuals, and previous meta-analysis reported mortality among patients co-infected with HCV was 1.35 times higher than that among patients with HIV-infection alone even in the highly active antiretroviral therapy (HAART) era [2]. In HIV-1/HCV co-infected patients, progression to liver cirrhosis and hepatocellular carcinoma (HCC) is faster than that in patients without HIV-1 infection [3]. Furthermore, the response to treatment with pegylated interferon (PEG-IFN) plus ribavirin (RBV) in HIV-positive patients with chronic HCV infection is poor (sustained virological response: SVR 19–40%), compared with patients infected with HCV alone (SVR 54–61%) [4]–[9].

The risk of HCV acquisition via heterosexual intercourse is estimated to be very low [10]. Recently, however, a high incidence of HCV seroconversion has been reported in HIV-1 infected men who have sex with men (MSM) [11]–[13]. These results suggest that new HCV infection can be a potential future problem in the clinical management of HIV-1 infected patients. On the other hand, a favorable response to treatment with PEG-IFN plus RBV for acute hepatitis C (AHC) relative to that for chronic one has been reported in HCV-infected (SVR 85–98%) [14], [15] and HIV/HCV co-infected patients (SVR 60–80%) [16], [17]. In this regard, the recent guidelines recommend PEG-IFN plus RBV treatment for AHC in HIV-1 co-infected patients [18]–[20]. However, data of AHC among HIV-1 infected patients is still limited, especially from Asian countries.

The response to treatment with PEG-IFN plus RBV is closely associated with the interleukin-28B (IL-28B) genotype, which encodes interferon-λ3 (IFN-λ3), in chronic HCV hepatitis, even in HIV-1 co-infected cases [21]–[23]. Furthermore, HCV mono-infected individuals with favorable IL-28B genotype (CC at rs12979860, TT at rs8099917) seem to achieve spontaneous clearance of HCV compared to those with non-favorable genotypes [21], [22], [24], [25]. To our knowledge, there are no studies on the effect of IL-28B genotype on the natural course and response to treatment of AHC in HIV-1 infected individuals in Asian population [24], [26], [27].

**Figure 1:**
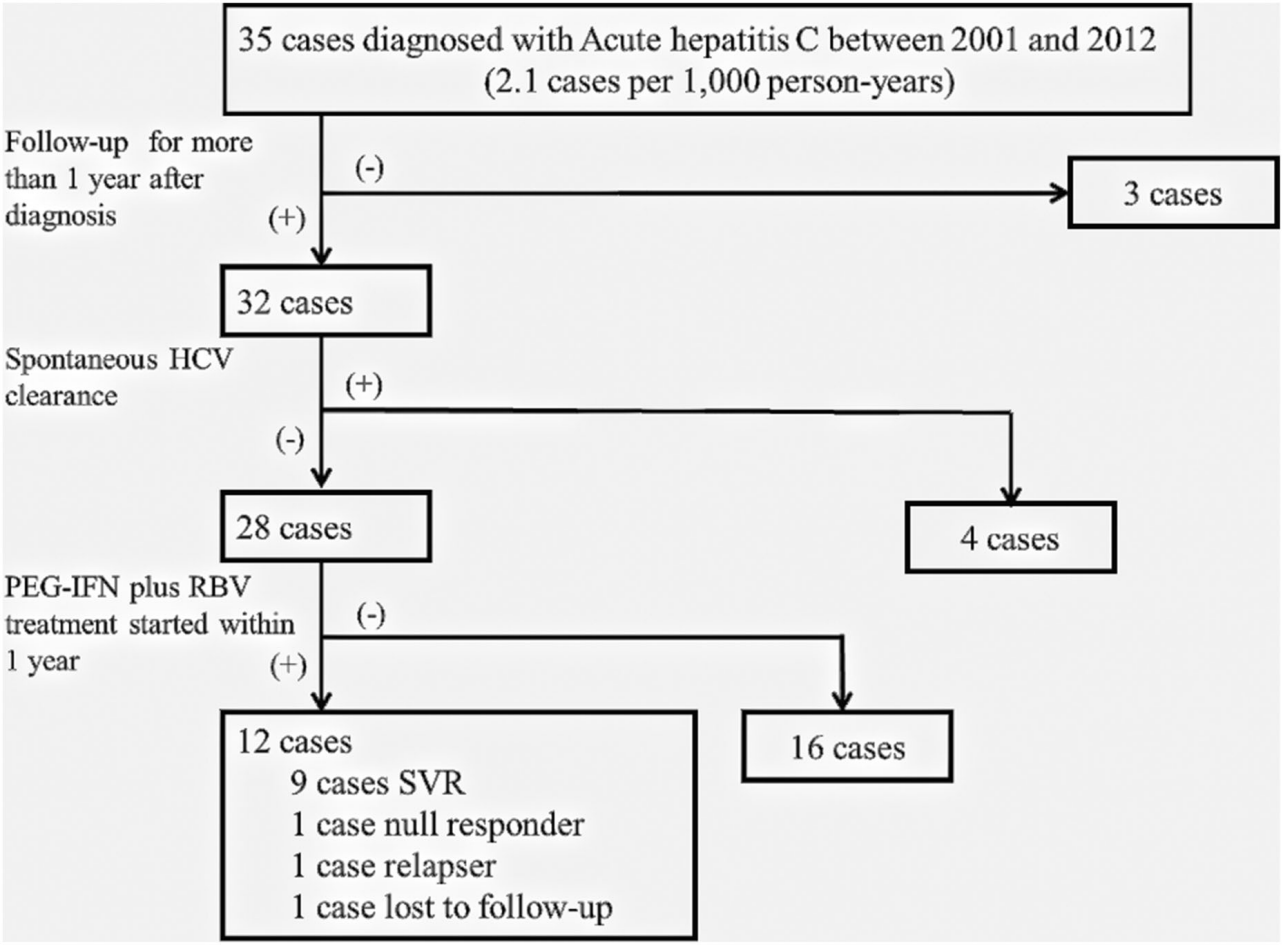
Patient enrollment process. Acute hepatitis C (AHC) was defined as elevation of alanine transaminase (ALT) >100 IU/L accompanied by seroconversion of anti-hepatitis C virus (HCV) antibody. Three patients could not be followed up for 1 year after diagnosis and were excluded from further analysis. HCV cleared spontaneously in 4 cases. PEG-IFN plus RBV treatment was initiated within 1 year of diagnosis of AHC in 12 out of 28 patients who did not show spontaneous clearance. One patient with missing treatment data following transfer to another clinic about two weeks after initiation of IFN plus RBV, was excluded from analysis related to the effect of PEG-IFN plus RBV. PEG-IFN: pegylated interferon, RBV: ribavirin.

In the last 12 years, 35 patients with HIV-1 infection were diagnosed with AHC in our hospital. In the present retrospective study, we report the results of analysis of data of 32 of these cases, and discuss the factors associated with spontaneous HCV clearance and response to treatment with PEG-IFN plus RBV (Fig. 1).

## Methods

### Study Design

This single-center retrospective cohort study was conducted in accordance with the ethical principles of the Declaration of Helsinki and of Good Clinical Practice. The ethics committee of National Center for Global Health and Medicine approved the study. All patients provided written informed consent.

### Study Participants

The medical records of HIV-1 infected patients in our institution, the largest HIV clinic in Japan, admitted and treated between January 2001 and December 2012, were retrospectively reviewed. AHC was defined according to the following criteria; elevation of alanine transferase (ALT) >100 IU/L accompanied by seroconversion of anti-HCV antibody, and exclusion of other causes (e.g., acute hepatitis B and drug-induced hepatitis). Patients who were lost to follow-up within 1 year from the diagnosis of AHC were excluded from the analysis since they could not be assessed for clinical presentation including spontaneous clearance. Spontaneous clearance was defined as a decrease in HCV RNA to undetectable level without treatment within one year from the diagnosis and remaining as such thereafter. For patients receiving PEG-IFN plus RBV treatment, we assessed the SVR rate. SVR was defined as continued undetectable HCV RNA at 24 weeks after completion of therapy. Baseline characteristics, status of HIV-1 infection, history of injecting drug usage (IDU), symptoms related to AHC (fatigue and jaundice), laboratory data abnormalities from AHC (ALT, T-bil), treatment of HCV infection and histological findings of liver biopsy, where available, and were collected from the medical records. We compared these variables between patients with and without spontaneous clearance of HCV and between responders and non-responders to treatment with PEG-IFN plus RBV.

### HCV Analysis

For each patient, titers of anti-HCV antibody were measured by a third-generation Latex aggregation assay (Ortho HCV Ab LPIA test III, Ortho Clinical Diagnostics, NJ) at first visit to our hospital and at diagnosis of AHC. Serum HCV RNA at each time point was extracted automatically (Cobas Ampliprep, Roche InVitro Diagnostics, Switzerland). Thereafter, cDNA was prepared and its titer was measured by quantitative polymerase chain reaction (Cobas TaqMan 48, Roche In Vitro Diagnostics). Direct sequencing was performed using DNA probe assay by ABI PRISM 3100 (Applied Biosystems, Foster City, CA). Finally, the genotype was determined from the amino acid sequences of 5 –untranslated region [28].

### Genotyping of IL-28b Alleles

Genomic DNA was isolated from peripheral blood mononuclear cells, using QIAamp DNA Mini Kit (Qiagen, Hilden, Germany). SNPs, rs12979860, and rs 8099917 were genotyped, using the TaqMan Drug Metabolism Assays by ABI PRISM 7900 HT sequence detection system (Applied Biosystems) according to the instructions provided by the manufacturer. The researchers responsible for genotyping were blinded to clinical data of the patients.

### Statistical Analysis

The patients’ characteristics and results of differences in viral clearance and virological response were compared using chi-square test (for qualitative variables) or Mann-Whitney U-test (for quantitative variables). Statistical significance was defined at two-sided *p* value of <0.05. All statistical analyses were performed with The Statistical Package for Social Sciences Version 21 (SPSS Inc, Chicago, IL).

## Results

### Patient Enrollment

A total of 35 patients were diagnosed with AHC during the study period. The incidence of AHC was 2.1 cases per 1,000 person-years. Three patients who were lost to follow-up within 1 year after diagnosis of AHC were excluded from the analysis. No deaths or fulminant hepatitis were recorded during the study period. Spontaneous HCV clearance was achieved by 4 patients, including 2 patients in whom HCV clearance was achieved within 3 months of diagnosis of AHC. The median time between diagnosis of AHC and HCV clearance was 11 weeks (range, 7–31 weeks). Among the 28 patients who did not show spontaneous HCV clearance, treatment with PEG-IFN plus RBV was initiated within 6 months of diagnosis in 9 patients and between 6 and 12 months of diagnosis of AHC in 3 patients (6.1, 6.4 and 6.9 months, respectively), whereas treatment was not initiated in the remaining 16 patients due to cost (n = 7) or other comorbidity (depression, history of epilepsy) (**Fig. 1**).

### Patients’ Characteristics and Clinical Presentations of AHC

The characteristics and clinical presentation of AHC patients are listed in **Tables 1** and **2**, respectively. All patients were Japanese men, including 31 (96.9%) MSM. Twenty nine patients (90.6%) received antiretroviral therapy (ART) and HIV-RNA was well suppressed in these patients. Four patients (12.5%) had a history of IDU, whereas none had a history of occupational exposure to HCV or blood transfusion. Although there was no significant difference between patients with and without spontaneous HCV clearance, the IL-28B CC+TT genotype was rs12979860 and rs8099917 in all 4 patients who showed spontaneous clearance and none of the patients with IL-28B CT+TG genotype showed spontaneous clearance (**Table 1**).

**Table 1:** Characteristics of AHC patients (n = 32).

	All patients(n = 32)	Spontaneousclearance (n = 4)	Non-spontaneousclearance (n = 28)	P-value
Age (years)	40 [30–58]	44 [37–56]	40 [30–58]	0.361
Male sex	32 (100)	4 (100)	28 (100)	-
Men who have sex with men	31(96.9)	4 (100)	27 (96.4)	1.000
IL-28B genotypes (rs12979860+rs8099917)				
CC+TT genotype	26 (81.2)	4 (100)	22 (78.6)	0.416
CT+TG genotype	6 (18.8)	none	6 (21.4)	-
TT+GG genotype	none	none	none	-
Injecting drug users	4 (12.5)	none	4 (14.3)	1.000
Received ART at diagnosis	29 (90.6)	4 (100)	25 (89.3)	1.000
CD4 count at diagnosis (cells/µL)	420 [167–824]	317 [184–616]	424 [167–824]	0.424
HIV-RNA at diagnosis (copies/mL)	UD [UD−9.4×10^4^]	50 [UD-50]	42.5 [UD-9.4×10^4^]	0.737

Data are number (%) of patients or median [range].

ART, antiretroviral therapy; UD, undetectable.

**Table 2:** Clinical presentation of AHC patients (n = 32).

	All patients (n = 32)	Spontaneous clearance (n = 4)	Non-spontaneous clearance (n = 28)	P-value
No symptoms	24 (75)	1 (25)	23 (82.1)	-
Symptoms	8 (25)	3 (75)	5 (17.9)	0.039
Fatigue	8 (25)	3 (75)	5 (17.9)	-
Jaundice	2 (6.25)	1 (25)	1(3.6)	-
Peak Alanine transaminase level (IU/L)	661 [117–2194]	707 [1237–2126]	614 [117–2194]	0.072
Peak total bilirubin level (mg/dL)	1.9 [0.7–17.0]	9.8 [4.2–17.0]	1.6 [0.7–6.8]	0.002
HCV genotype				
1a	1/27 (3.7)	None	1/2 (4.3)	-
1b	19/27 (70.4)	3/4 (75)	16/23 (69.6)	-
2a	4/27 (14.8)	1/4 (25)	3/23 (13)	-
2b	3/27 (11.1)	None	3/23 (13)	-
Not available	5	None	5	
HCV-RNA at diagnosis (Log IU/mL)	6.6 [1.9–7.8]¶	6.6 [4.9–6.8]†	6.6 [1.9–7.8]‡	0.594
Latency to HCV clearance (wks)*	-	11 [7]–[31]	-	-

Data are number (%) of patients or median [range] values.

*Time between AHC diagnosis and HCV clearance (weeks).

¶ Data of 6 patients not available for analysis.

† Data of 5 patients not available for analysis.

‡ Data of 1 patient not available for analysis.

The majority of patients with AHC (24/32, 75%) were asymptomatic at the onset of AHC. High ALT was identified incidentally at routine visit for HIV-1 infection, with subsequent tests confirming the diagnosis of AHC. Compared to patients who did not show spontaneous clearance, patients with spontaneous clearance showed more severe clinical presentation of hepatitis (symptomatic, with higher serum total bilirubin and ALT value at diagnosis). The most frequent HCV genotype was 1b (70.4%). At diagnosis, HCV RNA was higher than 5.0 LC/mL in 24 out of 26 patients (**Table 2**).

### Response to Treatment with PEG-IFN Plus RBV

Treatment with PEG-IFN plus RBV was initiated in 12 patients within 1 year of diagnosis of AHC (median interval from AHC diagnosis, 3.2 months). We assessed the response to treatment in only 11 patients; the other patient was lost to follow-up within two weeks of treatment initiation (**Fig. 1**, **Table 3**). SVR was achieved in 9 of 11 patients (81.8%) despite the high incidence of HCV genotype 1b and high viral load.

**Table 3:** Comparison of patients of the SVR and non-SVR groups.

	All patients (n = 11)	SVR (n = 9)	Non-SVR (n = 2)
Age (years)	38 [30–58]	38 [30–48]	52 [47–58]
Male sex	11 (100)	9 (100)	2 (100)
Men who have sex with men	11 (100)	9 (100)	2 (100)
IL-28B genotype			
CC+TT genotype	9 (81.8)	7 (77.8)	2 (100)
CT+TG genotype	2 (18.2)	2 (22.2)	None
Injecting drug users	1 (9.1)	None	1 (50)
Received ART before treatment	10 (90.9)	8 (88.9)	2 (100)
CD4 count before treatment (cells/µL)	382 [230–655]	440 [272–655]	238 [254–278]
HIV-RNA before treatment (copies/mL)	UD [UD-3.3×10^4^]	UD [UD-3.3×10^4^]	UD [305–610]
HCV genotype			
1b	10 (90.9)	8 (88.9)	2 (100)
2a	1 (9.1)	1 (11.1)	None
HCV-RNA before treatment (Log IU/mL)	6.3 [3.3–7.8]	6.3 [5–7.8]	5.7 [3.6–8.0]
Latency to AHC diagnosis (months)*	3.2 [0.9–6.9]	3.2 [0.9–6.9]	4.4 [3.7–5.1]
Duration of PEG-IFN+RBV therapy (wks)	43 [11–72]	43 [11–72]	36 [11–60]
Latency to HCV clearance (wks)¶	-	8 [3]–[16]	-
RVR	3 (27.2)	3 (33.3)	None
EVR	7 (63.6)	6 (66.7)	1 (50)
Histopathology positive for liver fibrosis			
F0	3/6	3	0
F1	2/6	1	1
F2	1/6	0	1
F3	0	0	0

Data are number (%) of patients or median [range] values.

*Time between AHC diagnosis and initiation of therapy (months).

¶ Time between initiation of therapy and HCV clearance (weeks).

ART, antiretroviral therapy; UD, undetectable; PEG-IFN+RBV, pegylated interferon plus ribavirin; SVR, sustained viral response; EVR, early viral response; RVR, rapid viral response.

Two patients did not achieve SVR. Both patients were infected by genotype 1b with high viral load, and treatment was initiated within 6 months of diagnosis. One achieved viral clearance within 12 weeks (early virological response: EVR) but showed viral rebound at 15 weeks after completion of the treatment (relapser), whereas viral clearance was not achieved during treatment in the other patient (null-responder). Both patients were relatively older and their CD4 counts were lower, compared to those with SVR, although statistical analysis was not performed due to the small number of cases. In patients with SVR, the median time between initiation of therapy and clearance of HCV was 8 weeks (range, 3–16 weeks). Surprisingly, both patients with IL-28B CT+TG alleles achieved SVR despite genotype 1b and high viral load, although we could not compare the SVR rate among different genotypes since only one patient was infected with genotype 2a in this study.

### Histological Findings of AHC in HIV-1 Co-infected Patients

**Figure 2:**
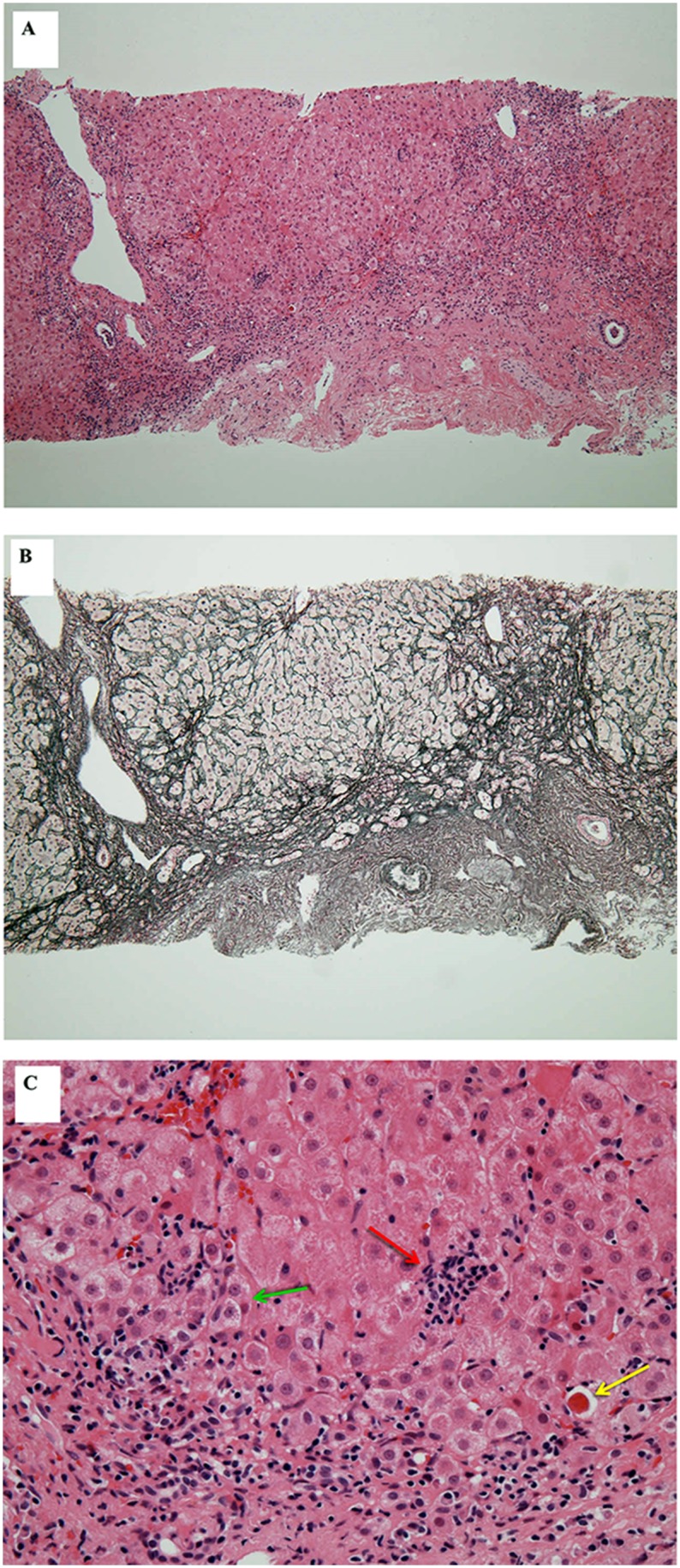
Histological findings in needle liver biopsy specimen from the patient who showed null-response (Table 3). The pre-treatment biopsy specimen obtained at 13 weeks after AHC diagnosis showed stage 2 fibrosis (F2) according to the classification of chronic hepatitis C (New Inuyama Classification). (A and B) Formation of bridging fibrosis by fibrous and cellular expansion in the portal tract. (C) Magnified view showing centrilobular piece-meal necrosis (green arrow), acid folic body (yellow arrow) and spotty necrosis (red arrow). (A) Hematoxylin-eosin stain, x100, (B) Silver impregnation stain, x100, (C) Hematoxylin-eosin stain, x400. PEG-IFN: pegylated interferon, RBV: ribavirin, AHC: acute C hepatitis.

HBs antigen was negative and ALT was within the normal range in the year preceding AHC in all 6 patients, whereas HBs Ab and/or HBc Ab was positive in 5 patients. No pre-existing factors of liver fibrosis other than HIV infection were evident before AHC. Liver biopsy was performed in 6 patients before treatment with PEG-IFN plus RBV. The median interval between diagnosis of AHC and biopsy was 4.3 months (range, 3.3–6.1 months). Fibrotic changes were confirmed in 3 cases by hematoxylin-eosin staining and silver impregnation staining (**Fig. 2**, **Table 3**). These lesions were paler-staining by Victoria Blue stain, indicating that the fibrotic areas did not reflect chronic changes.

## Discussion

In the present study, we identified 35 cases of AHC during the study period and nearly all such patients (34/35) were MSM, and the most frequent HCV genotype was 1b (19/27). These findings are consistent with previous reports from other countries [11]–[13]. In this regard, a high incidence of HCV seroconversion in HIV-1 infected MSM was reported recently by two separate groups [11]–[13]. The same studies also reported that genotype 1b was the major genotype among their patients [11]–[13], and that HCV infection was frequently not detected during the acute phase and diagnosed only at the chronic stage mainly due to the lack of symptoms.

Similar to the previous reports on AHC, 75% of our cases were asymptomatic, and only 6.3% of the study population showed mild elevation of serum ALT (100 IU/L < ALT <150 IU/L). In this regard, ALT elevation during acute HCV infection is often relatively transient, and therefore could be easily missed during routine clinical care. The need of regular screening for anti-HCV antibody in HIV-1 infected MSM is controversial, and the recommendations are different in guidelines from different developed countries [29], [30]. Our results emphasize the importance of regular ALT monitoring and HCV re-screening at the time of mild ALT elevation during follow-up, especially in high-risk populations such as sexually active MSM.

There are few reports on the relationship between IL-28B CC+TT genotype and spontaneous clearance of HCV [21], [31]. In the present study, spontaneous HCV clearance was seen in 4 out of 26 patients with IL-28B CC+TT genotype, whereas no spontaneous HCV clearance was seen in all 6 patients with IL-28B CT+TG genotype. Although this difference could not be confirmed to be statistically significant due to the small number of patients (4 patients), this is, to our knowledge, the first report on the relation between IL-28B and spontaneous HCV clearance during AHC in HIV-1 co-infected patients in Asian population. Our study also showed that the severity of clinical symptoms was an important factor related to spontaneous HCV clearance. Further investigation is needed for a better understanding of the pathogenesis of AHC, especially factors involved in spontaneous clearance.

The use of PEG-IFN plus RBV treatment for AHC within 6 months of diagnosis is now recommended for HIV-1 co-infected cases [17]–[19] although data on the response of HIV-1 infected individuals with AHC to the PEG-IFN plus RBV remain limited. One study reported spontaneous clearance of HCV between 6 and 12 months of diagnosis [32]. In this regard, it is sometimes difficult in the clinical setting to start PEG-IFN plus RBV treatment within 6 months of diagnosis because some patients have comorbidities and complications other than HIV and HCV. In our analysis, 9 of 11 patients (81.8%), including 2 patients whose treatment was initiated between 6 and 12 months of diagnosis, achieved SVR despite high rate of genotype 1b infection (SVR 90.0% among those with genotype 1b virus). Furthermore, HCV genotype 1b-infected patients carrying the IL-28B CT+TG genotype (n = 2), which is a predictor of poor response to the treatment of chronic HCV infection, achieved SVR. These results emphasize the advantage of the PEG-IFN plus RBV treatment for AHC.

Little is known about the progression of AHC to liver fibrosis in patients with HIV/HCV co-infection [33], although rapid progression of liver fibrosis during the chronic phase is well recognized [3]. Fierer et al. [34] reported that the development of fibrosis occurs even in the acute phase of HCV infection in HIV-Infected men. In the present study with limited cross-sectional analysis of liver biopsies after AHC, fibrosis was detected in 3 out of 6 cases, which is consistent with the above report of Fierer et al. [34]. Moreover, SVR was not achieved in 2 out of 3 patients who showed liver fibrosis, whereas the other 3 patients without fibrosis achieved SVR (**Table 3**). These results emphasize the clinical importance of early diagnosis and early treatment for AHC in HIV-1 infected individuals.

In conclusion, the potential of AHC should always be considered in HIV-1 infected MSM, even in asymptomatic case, who present with mild ALT elevation. Favorable response can be expected if anti-HCV treatment is initiated during the early phase. Further investigation is needed to determine the predictor(s) of spontaneous HCV clearance, appropriate timing of treatment initiation, and duration of treatment.
